# Clinical Scaleup of Humanized AnnA1 Antibody Yielded Unexpected High Reticuloendothelial (RES) Uptake in Mice

**DOI:** 10.3390/antib14010014

**Published:** 2025-02-06

**Authors:** Lu Lucy Xu, Satyendra Kumar Singh, Chelsea Nayback, Abdullah Metebi, Dalen Agnew, Tim Buss, Jan Schnitzer, Kurt R. Zinn

**Affiliations:** 1 Biomedical Engineering, Michigan State University, East Lansing, MI 48824, USA; xulu2@msu.edu (L.L.X.); singhs54@msu.edu (S.K.S.); 2 Institute for Quantitative Health Science and Engineering, Michigan State University, East Lansing, MI 48824, USA; naybackc@msu.edu (C.N.); metebiab@msu.edu (A.M.); 3 College of Human Medicine, Michigan State University, East Lansing, MI 48824, USA; 4 Department of Pathobiology and Diagnostic Investigation, College of Veterinary Medicine, Michigan State University, East Lansing, MI 48824, USA; agnewd@msu.edu; 5 Proteogenomics Research Institute for Systems Medicine (PRISM), La Jolle, CA 92037, USA; tbuss@prism-sd.org (T.B.); jschnitzer@prism-sd.org (J.S.); 6 Department of Radiology, College of Human Medicine, Michigan State University, East Lansing, MI 48824, USA; 7 Department of Small Animal Clinical Sciences, College of Veterinary Medicine, Michigan State University, East Lansing, MI 48824, USA

**Keywords:** reticuloendothelial system, mouse model, antibody, immunotherapy, cancer therapeutics, toxicity, annexin, IgG

## Abstract

Background/Objectives: A mouse antibody directed against truncated Annexin A1 showed high tumor retention in pre-clinical cancer models and was approved by the National Cancer Institute Experimental Therapeutics (NExT) program for humanization and large batch cGMP production for toxicology and clinical trials. In this process, a contractor for Leidos accidentally produced a mutated version of humanized AnnA1 (hAnnA1-mut) with a single nucleotide deletion in the terminal Fc coding region that increased the translated size by eight amino acids with random alterations in the final twenty-four amino acids. We investigated the tissue distribution of hAnnA1-mut, hAnnA1, mAnnA1, and isotope-matched human IgG1 under various injection and conjugation conditions with C57BL/6, FVB, and BALB/c nude mice strains. Methods: Biodistribution studies were performed 24 h after injection of Tc-99m-HYNIC radiolabeled antibodies (purity > 98%). Non-reducing gel electrophoresis studies were conducted with IR680 labeled antibodies incubated with various mouse sera. Results: Our results showed that Tc-99m-HYNIC-hAnnA1 had low spleen and liver retention not statistically different from Tc-99m-HYNIC-IgG1 and Tc-99m-HYNIC-mAnnA1, with corresponding higher blood levels; however, Tc-99m-HYNIC-hAnnA1-mut had high levels in the spleen and liver with differences identified among the mouse strains, radiolabeling conditions, and injection routes. Histopathology showed no morphological change in the liver or spleen from any conditions. Gel electrophoresis showed an upward shift of hAnnA1-mut, consistent with the binding of blood serum protein. Conclusions: The changes in the Fc region of hAnnA1-mut led to higher liver and spleen uptake, suggesting the antibody’s recognition by the innate immune system (likely complement protein binding) and subsequent clearance. Future clinical translation using hAnnA1 and other antibodies needs to limit protein modifications that could drastically reduce blood clearance.

## 1. Introduction

Annexins are cytosolic 37 kDa phospholipid-binding proteins with conserved 3D structures that bind acidic phospholipids in cellular membranes at elevated Ca^2+^ levels. They function to organize membrane lipids, facilitate cellular membrane transport, and display extracellular activities [1,2]. Annexin A1, a phospholipase inhibitor protein, is a member of this family that mediates the anti-inflammatory activity of glucocorticoids first discovered in neuroendocrine cells [2,3]. Annexin A1 is most prominent in differentiated cells (i.e., macrophages, neutrophils) and various forms of cancers (i.e., fibrosarcoma, myeloma, breast, pancreatic) [4,5,6,7]. It has been reported to be a cytosolic protein, but stress at the cellular level may result in the externalization of the protein during cell death [8]. A post-translationally truncated version of Annexin A1 cleaved at Lys26 34 kDa in size localized to the caveolae of endothelium from multiple human and rat primary and metastatic tumors (i.e., breast, kidney, liver, lung, brain, and prostate), but not in normal tissue [9]; therefore, targets to Annexin A1 should not be present on the surface membrane of healthy cells unless there is damage to cells that caused externalization of the protein or if modifications have occurred to the targeting agent.

Caveolae are transport vesicles that line the luminal surface of vascular endothelial cells directly in contact with blood and can transport molecules across the endothelium [10]. An antibody targeting the truncated Annexin A1 protein was shown to rapidly pump across the tumor endothelium, reaching >100 fold more than other antibodies and exceeding peak blood fluorescence signal within one hour. This trans-vascular pumping mechanism required expression of both Annexin A1 and caveolin 1 [11]. The delivery of monoclonal antibodies that are actively pumped into tumors through the vascular endothelium could potentially enhance the targeting of disseminated cancers that are otherwise unsuitable for surgery. This is particularly relevant for ovarian cancer, characterized by a high rate of recurrence and scattered peritoneal metastasis due to the disease’s non-specific symptoms and delayed diagnosis [12]. Conjugating an antibody that can be actively pumped through the tumor endothelium with a radioisotope may add enhanced tumor-killing potential and provide an even more promising therapeutic target to elicit direct DNA damage leading to cell death [13]. In this study, we are testing modifications to antibodies targeting the truncated version of Annexin A1 localized in caveolae of the endothelium.

Our studies were initiated after the NExT program produced hAnnA1 and showed unexpectedly high liver and spleen uptake in mice. The concern was reduced blood levels of hAnnA1 available for tumor targeting and potential toxicity to off-target tissues, making clinical applications of the antibody less effective. The studies reported here were all completed before we knew the NexT program produced hAnnA1 was actually mutated and what we now refer to as hAnnA1-mut, an antibody with random 24 amino acids at the Fc end. We initially thought the problem with liver and spleen uptake was related to differences in glycosylation due to the scaled-up production of hAnnA1 by the NExT program. This study comprehensively evaluated how alterations in the constant region of the Fc terminus of the hAnnA1 antibody changed its biodistribution in immune-competent mice. Several mouse strains (FVB, C57BL/6, BALB/c nude), injection routes (IV vs. IP), and chelator:antibody molar conjugation ratios (1:1 vs. 6:1) were compared for the hAnnA1-mut antibody versus controls.

## 2. Materials and Methods

### 2.1. Antibody Growth

Four antibodies were compared in this study, which included human IgG1 isotype control (Cat #0151K-01, Southern Biotech, Birmingham, AL, USA), mouse version (mAnnA1) (PRISM, La Jolla, CA, USA), and two humanized versions (hAnnA1 and hAnnA1-mut). Human and mouse antibodies differed in their isotypes. hAnnA1 was derived from human IgG1 and mAnnA1 was created from mouse monoclonal IgG2b. A human IgG1 Fc region was attached when mAnnA1 was humanized to create hAnnA1.

#### 2.1.1. hAnnA1 Antibody Production

The mouse monoclonal antibody, mAnnA1, was generated by the Proteogenomics Research Institute for Systems Medicine (La Jolla, CA, USA) using conventional hybridoma methodology [14]. The complementarity determining regions (CDRs) were grafted to the framework of an appropriate germline human antibody for humanization to construct hAnnA1 [15,16,17]. To restore the affinity of hAnnA1 to the same level as mAnnA1, a single framework back mutation was introduced into the VH and Vk regions. hAnnA1 sequence was provided to contractors from the NCI NExT program contractors for cGMP production. This resulted in a mistake when generating the heavy chain expression vector, which resulted in hAnnA1-mut used in this study. Unfortunately, the mistake was reported to MSU only after all experiments conducted in this study were finished, as MSU investigators were trying to understand the differences in liver and spleen uptake between PRISM-produced hAnnA1 and the hAnnA1 antibody provided by Leidos (now called hAnnA1-mut), the contractor for the NExT program.

#### 2.1.2. hAnnA1 Antibody Purification

The antibodies obtained from PRISM were harvested by spinning out the cell culture, and the supernatant was filtered (0.2 microns). This was passed over a protein G column (Cytiva) on the AKTA fast protein liquid chromatography (Cytiva, Marlborough, MA, USA) and washed with PBS (25 column volumes) until a stable baseline followed by elution with 0.1 M glycine, pH 2.6. The peak was dialyzed against PBS and sent to analytics for size exclusion chromatography (SEC) polishing. The final antibody was ~98% monomer, and A280 for 1 mg/mL of hAnnA1 was 1.53.

In this study, an hAnnA1-mut antibody was produced from CHO cells expressing stable hAnnA1 where one nucleotide deletion occurred that led to 24 base pairs of nucleotide addition in the coding sequence. hAnnA1-mut antibody involved urea step for purification by Leidos (Frederick, MD, USA) and Aragen (Nacharam, Hyderabad, India); however, hAnnA1 (with correct sequence) was produced in CHO cells with transient hAnnA1 expression (correct sequence) purified by PRISM without the urea step of purification (hAnnA1).

#### 2.1.3. DNA and Amino Acid Sequencing

An aliquot of the hAnnA1 master cell bank from Leidos was analyzed by WuXi AppTec to confirm the transgene sequence as part of the normal release testing of GMP products for the FDA. This release testing identified hAnnA1-mut.

### 2.2. Tc-99m-HYNIC Antibody Labeling

#### 2.2.1. HYNIC-Antibody Conjugation

N-succinimidyl-hydrazino nicotinate (HYNIC) was resuspended in Na_2_HPO_4_ buffer (150 mM, pH = 7.8) and added to the antibodies at a molar ratio 6:1 or 1:1 (HYNIC:Ab) and incubated at room temperature in the dark for 1 h on a rocker. The conjugated HYNIC-AnnA1/ isotype IgG1 was dialyzed overnight at 1 L PBS (1×) buffer to remove any unconjugated HYNIC using Slide-A-Lyzer Dialysis Cassette for 10,000 MWCO, 0.5–3 mL capacity (Thermo-Fisher, Waltham, MA, USA). After dialysis, HYNIC-antibody conjugates were collected into 1.5 mL tubes and stored at 4 °C until use.

#### 2.2.2. Radiolabelling

For radiolabeling, 1.85 GBq Tc-99m pertechnetate in ~1.0 mL (Cardinal Health, Flint, MI, USA) was added to a Tricine/SnCl_2_ (36 mg/0.05 mg) kit and incubated at room temperature for 15–30 min. Next, Tc-99m/Tricine/SnCl_2_ mixture was added to the HYNIC-hAnnA1/hAnnA1-mut/mAnnA1/IgG conjugate and incubated for 45 min at room temperature at 500 rpm, and shaken in the dark. Tc-99m radiolabeled antibodies were purified using a G25 Sephadex resin column, and the purity (Tc-99m bound) was checked by iTLC using PBS or 2-butanone (MEK) as the mobile phase. In this study, the purity of injected radiolabeled antibodies was >98% for all experiments. After radiolabeling and purification, antibody concentrations were determined by using the Bradford assay to calculate specific activity. Radiolabeled antibodies were stored at 4 °C until injection.

### 2.3. Biodistribution

All animal studies were approved by the Institutional Animal Care and Use Committee (IACUC) of Michigan State University, USA, and all animal experiments were conducted according to IACUC guidelines (PROTO202300321). C57BL/6, FVB, and BALB/c nudes were obtained from Charles River (Wilmington, MA, USA) and used for biodistribution studies between 10 and 12 weeks. All strains were divided into 4 per group, with the exception of BALB/c nude mice with 6 per group. The sample size was determined using power analysis (two-tailed *t*-test, alpha = 0.05, power = 0.9, expected 60% differences in means, 5% relative standard deviation, 10% attrition). The primary outcome measure used for determining sample size was the % injected dose/gram of tissue (%ID/g) for the liver and spleen. The groups were randomized by weight using simple randomization. A total of 80 animals were used for the biodistributions. Single blinding was used throughout the process. The dose of Tc-99m-HYNIC-AnnA1/IgG was 1 mg/kg and was injected via intravenous (IV) and intraperitoneal (IP) routes. Mice were euthanized after 24 h on isoflurane. Different internal organs were collected, and activities in different organs were counted on a calibrated Gamma Counter (Wizard2 2480 Automatic Gamma Counter, Revvity, Waltham, MA, USA). Exclusion criteria included incomplete injections and % recovered dose <60%, established a priori.

### 2.4. IRDye680 Antibody Labeling

The antibody was diluted in PBS without sodium azide at a concentration of 1 mg/mL, and the pH was maintained at 8.5 using 1 M potassium phosphate buffer (pH 9). IRDye680 (LI-COR Biosciences, Lincoln, NE, USA) was labeled with different antibodies as per the manufacturer’s instructions. Briefly, a tube of IRDye680 dye was resuspended in 25 µL of dH_2_O and vortexed. The volume of dye added to our antibody solution was determined using 1003 divided by the molecular weight of the antibody (150 MW) according to the manufacturer’s specifications and mixed thoroughly. The mixture was allowed to react for 2 h at 20 °C, protected from light. The free dye was separated from the conjugated antibody using the 2 mL Zeba desalting column >7 kDa (Thermo-Fisher, Waltham, MA, USA).

### 2.5. Blood Sera Isolation

Whole blood was collected from mice and healthy individuals. The collected blood was allowed to clot by leaving it undisturbed for 30 min at room temperature. Vials were centrifuged at 3000 rpm for 10 min at 10 °C. The resulting supernatant was transferred to 100 μL aliquots and stored at −20 °C. The concentration of protein was determined using a Bradford assay.

### 2.6. Serum Binding Assay Using Gel Electrophoresis

The AnnA1 antibody serum binding assay was performed using NuPAGE Bis-Tris gel electrophoresis, as per the manufacturer’s instruction. Briefly, 25 μg of sample sera or positive control (AnnA1 antigen) were mixed with 2 μg of fluorescently labeled hAnnA1, hAnnA1-mut, mAnnA1, or IgG antibodies. These antigen-antibody mixtures were incubated for 30 min at 37 °C. Next, protein samples were mixed with 4× protein sample loading buffer (LI-COR Biosciences, Lincoln, NE, USA) and added to their respective wells in NuPAGE Bis-Tris gel. The gels were run at 200 V for ~3 h on ice. The gels were kept in the NuPAGR 15 well 8% mini-gel cassette (Thermo-Fisher, Waltham, MA, USA) and imaged using fluorescence (excitation = 675 nm, absorption= 720 nm) on an IVIS system (Spectrum model, Revvity, Waltham, MA, USA).

### 2.7. Histopathology

During biodistribution studies, liver and spleen tissues were collected and fixed in 10% formalin. The protocol-specific tissues from each animal were sectioned at approximately 5 um, stained with hematoxylin–eosin (H&E), and reviewed by a veterinary pathologist. Selected slides that were analyzed included those with the highest uptake from each group.

### 2.8. Statistical Analysis

The statistical analyses were conducted using GraphPad Prism 9.0 (La Jolla, CA, USA) and Microsoft Excel 2020 (Redmond, WA, USA). Data were presented as mean ± standard error of the mean (SEM). Statistical differences were determined by two-way analysis of variance (ANOVA). *p* < 0.05 was considered statistically significant.

## 3. Results

### 3.1. hAnnA1 Antibody Sequencing

**Figure 1:**
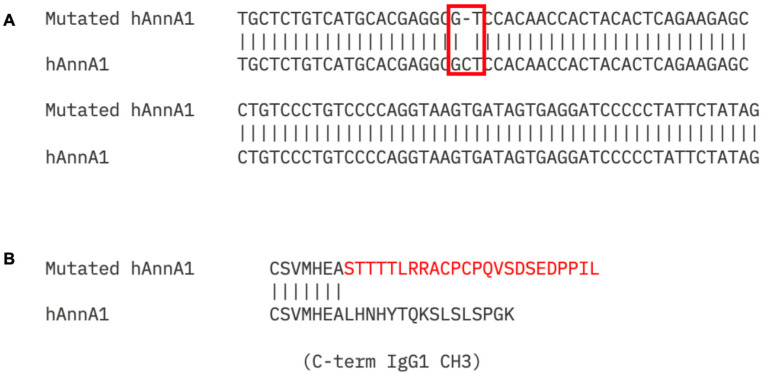
Nucleotide and amino acid sequence alignments for hAnnA1 and hAnnA1-mut. hAnnA1-mut was aligned with the hAnnA1 reference sequence and noticed a single nucleotide deletion towards the end of the heavy chain CH3 domain. This deletion results in a frameshift that results in 23 erroneous amino acids replacing the expected final 16 amino acids of the CH3. The bottom sequence shows the C terminal of the IgG1 CH3 domain. The top sequence (**A**) shows the location of the single nucleotide deletion with the correct sequence below. The translation (**B**) shows the effect of the resulting frameshift. The altered amino acids are highlighted in the red text.

After unexpected liver and spleen uptake was observed in initial uptake studies using hAnnA1-mut, antibody sequencing was conducted, comparing the original sequence to the one produced from the NexT program (hAnnA1-mut). The nucleotide sequence alignment showed a single nucleotide deletion in the sequence of hAnnA1 at position 1423 (red box), resulting in a frameshift mutation in the heavy chain CH3 region of the antibody to produce hAnnA1-mut antibody (Figure 1A). This mutation led to a 24 bp frameshift modification to the coding nucleotide, replacing the expected final 16 amino acids (Figure 1B). Further, this frameshift mutation resulted in an increase of eight amino acids (445 aa in hAnnA1 vs. 453 aa in hAnnA1-mut) and twenty-four base pairs (1338 bp in hAnnA1 vs. 1362 bp in hAnnA1-mut) as shown in Table 1. Thus, hAnnA1-mut had 24 amino acids at the Fc end that were random due to a nucleotide base pair deletion as compared with hAnnA1, which had eight fewer amino acids and the correct sequence for the final sixteen amino acids.

### 3.2. Effect of HYNIC Molar Conjugation Ratio on RES Uptake

**Figure 2:**
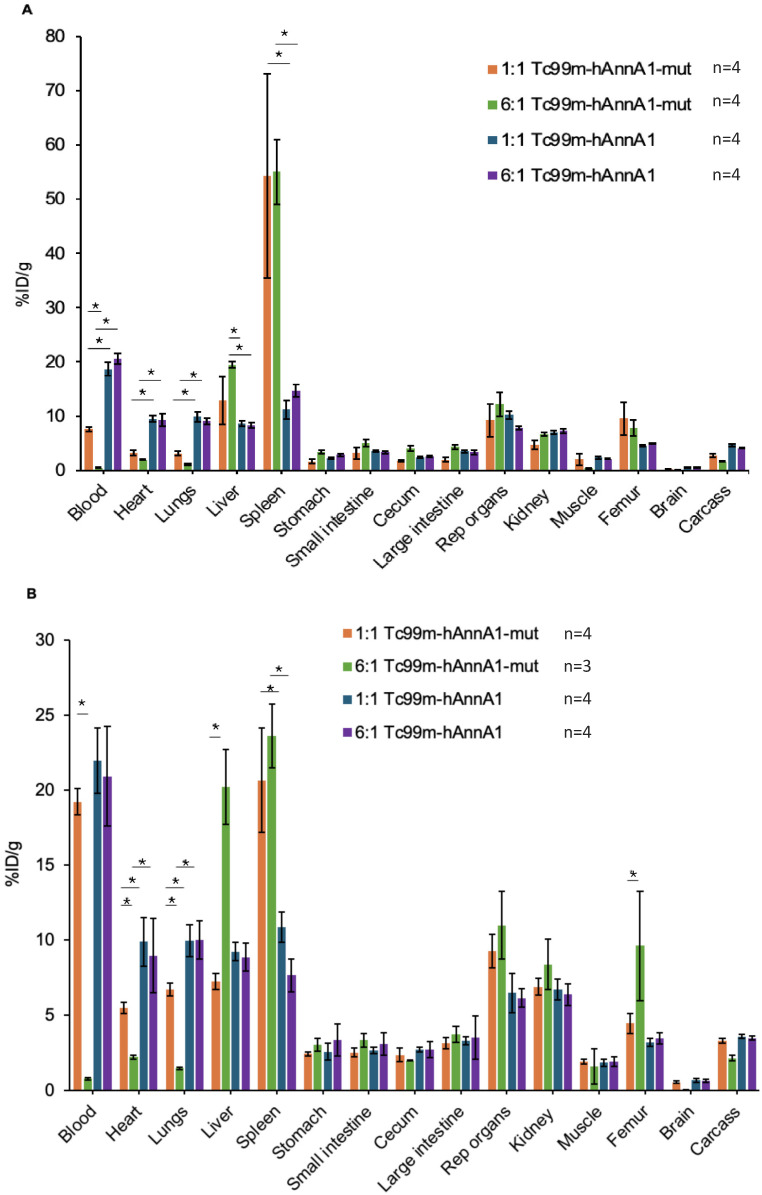
AnnA1 and HYNIC (chelator) conjugation ratio affects the AnnA1 antibody’s RES uptake. Bar graphs showing biodistribution of hAnnA1-mut and hAnnA1 antibody conjugated at 1:1 vs. 6:1 ratio (HYNIC: Ab) and after 24-h of injection in (**A**) C57BL/6 mice and (**B**) FVB mice. * *p* < 0.05.

There are reports of surface modifications to antibodies that could influence their pharmacokinetics and RES uptake [18,19,20]. In this study, we conjugated hAnnA1-mut and hAnnA1 antibodies with the HYNIC chelator at a molar ratio of 1:1 and 6:1 (HYNIC: Ab) to determine if there was a change to the biodistribution when altering conjugation ratios. After radiolabeling antibodies with Tc-99m, we IP injected Tc-99m-HYNIC-hAnnA1-mut or Tc-99m-HYNIC-hAnnA1 and performed biodistribution analysis 24 h following injection in C57BL/6 (Figure 2A) and FVB (Figure 2B) mice.

Our results demonstrated that in both mice strains, the amount of Tc-99m-HYNIC-hAnnA1-mut antibody in blood was significantly lower for the 6:1 molar conjugation ratio (0.5 ± 0.06 %ID/g for C57BL/6 and 0.5 ± 0.06 %ID/g FVB) compared with a 1:1 conjugation ratio (7.6 ± 0.4 %ID/g for C57BL/6 and 19.2 ± 0.9 %ID/g for FVB) (*p* < 0.05). For both strains of mice, there were no significant differences in Tc-99m-HYNIC-hAnnA1 antibody levels in blood between the 1:1 and 6:1 conjugation ratios. The reduced amounts of Tc-99m-HYNIC-hAnnA1-mut in blood correlated with significantly increased uptake in the liver and spleen compared with the Tc-99m-HYNIC-hAnnA1 antibody, which suggested that there was less Tc-99m-HYNIC-hAnnA1-mut available in the circulation when off-target RES uptake increased. This effect was even more prominent for the higher 6:1 conjugation ratio in FVB mice with significantly elevated levels of uptake in the liver (20.2 ± 2.5 %ID/g vs. 7.2 ± 0.5 %ID/g) and femur (9.6 ± 3.6 %ID/g vs. 4.5 ± 0.6 %ID/g) for the 6:1 conjugation vs. 1:1 conjugation, respectively. The differences in tissue distribution as related to molar conjugation ratios were not as apparent in C57BL/6 mice. These findings suggest that antibody-to-chelator conjugation ratio in combination with antibody alterations (e.g., Fc terminus difference of 24 amino acids) for hAnnA1-mut antibody plays an important role in biodistribution of Tc-99m-HYNIC-hAnnA1-mut antibody, especially in FVB mice.

### 3.3. RES Uptake in Mice Was Changed by Injection Route

**Figure 3:**
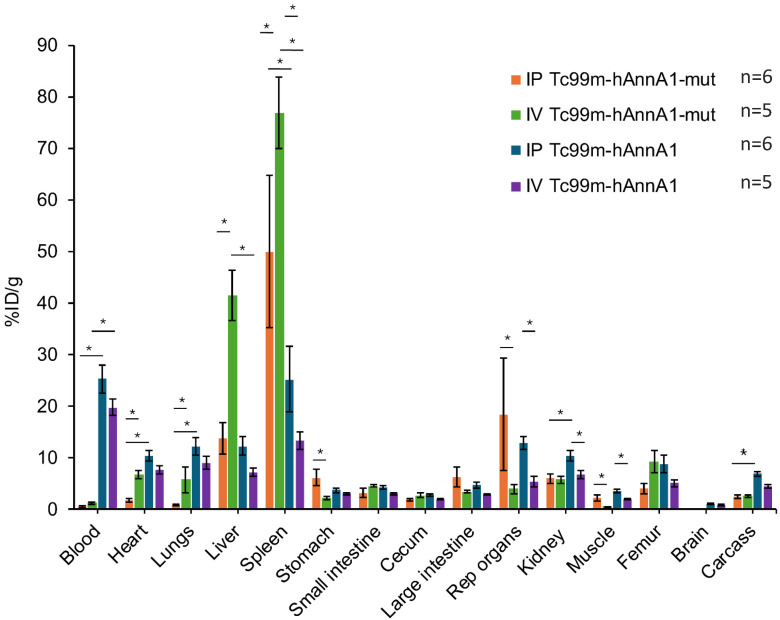
Effect of injection route on AnnA1 RES uptake. Bar graph comparing biodistribution of hAnnA1-mut and hAnnA1 antibody conjugated at 1:1 (HYNIC:Ab) and after 24 h of IP vs. IV Injection in BALB/c nude mice. * *p* < 0.05.

We have a strong interest in ovarian cancer and its disseminated states in the peritoneal cavity, with many of the therapies delivered through IP injection; therefore, we are curious about how different routes of injection could impact RES uptake. An IP route may be more effective and lead to rapid tumor uptake with less off-site targeting, including the liver and spleen; therefore, we evaluated whether the antibody injection route had any effects on RES uptake. We conjugated hAnnA1 and hAnnA1-mut antibodies at a molar ratio 1:1 (Ab:HYNIC), and after radiolabeling with Tc-99m, injected Tc-99m-HYNIC-hAnnA1, or Tc-99m-HYNIChAnnA1-mut through IP or IV routes in BALB/c nude mice (Figure 3). After 24 h of injection, we evaluated the biodistribution of both Tc-99m-HYNIC-hAnnA1 and Tc-99m-HYNIC-hAnnA1-mut antibodies. Our results showed that Tc-99m-HYNIC-hAnnA1-mut had a significantly higher uptake (*p* < 0.05) in the spleen and liver when injected IV (spleen = 77 ± 6.9 %ID/g, liver = 41.5 ± 4.9 %ID/g) compared with IP (spleen = 50 ± 14.7 %ID/g, liver = 13.8 ± 3.1 %ID/g). Tc-99m-HYNIC-hAnnA1 had a moderately higher uptake in the spleen when injected IP (25.3 ± 6.4 %ID/g) compared with IV (13.4 ± 1.7 %ID/g). In the liver, hAnnA1-mut with IV injection (41.5 ± 4.9 %ID/g) had a significantly higher uptake compared with IP injection (13.8 ± 3.1 %ID/g) (*p* < 0.05). There is also higher uptake for both Tc-99m-HYNIC-hAnnA1-mut (18.4 ± 10.9 %ID/g) and Tc-99m-HYNIC-hAnnA1 (12.9 ± 1.3 %ID/g) using IP injection in reproductive organs compared with IV injection for Tc-99m-HYNIC-hAnnA1-mut (4 ± 0.9 %ID/g) and Tc-99m-HYNIC-hAnnA1 (5.3 ± 1 %ID/g). The lower amount of uptake for Tc-99m-HYNIC-hAnnA1 in the RES organs correlated with the higher blood levels for both IV and IP injection, indicating more antibodies were available in circulation if it was not removed by the liver or spleen. These data suggest that IV injection results in a significantly higher uptake of Tc-99m-HYNIC-hAnnA1-mut in RES organs, while the differences were not found for Tc-99m-HYNIC-hAnnA1. A possible explanation for the higher RES uptake with IV injection may be due to the rapid increase in antibody blood concentration leading to a greater concentration gradient compared with a more gradual increase with IP injection, where it first needs to cross into the circulation.

### 3.4. Differential Uptake of Annexin A1 Antibody in FVB, C57BL/6, and BALB/c Nude Mice Strains

**Figure 4:**
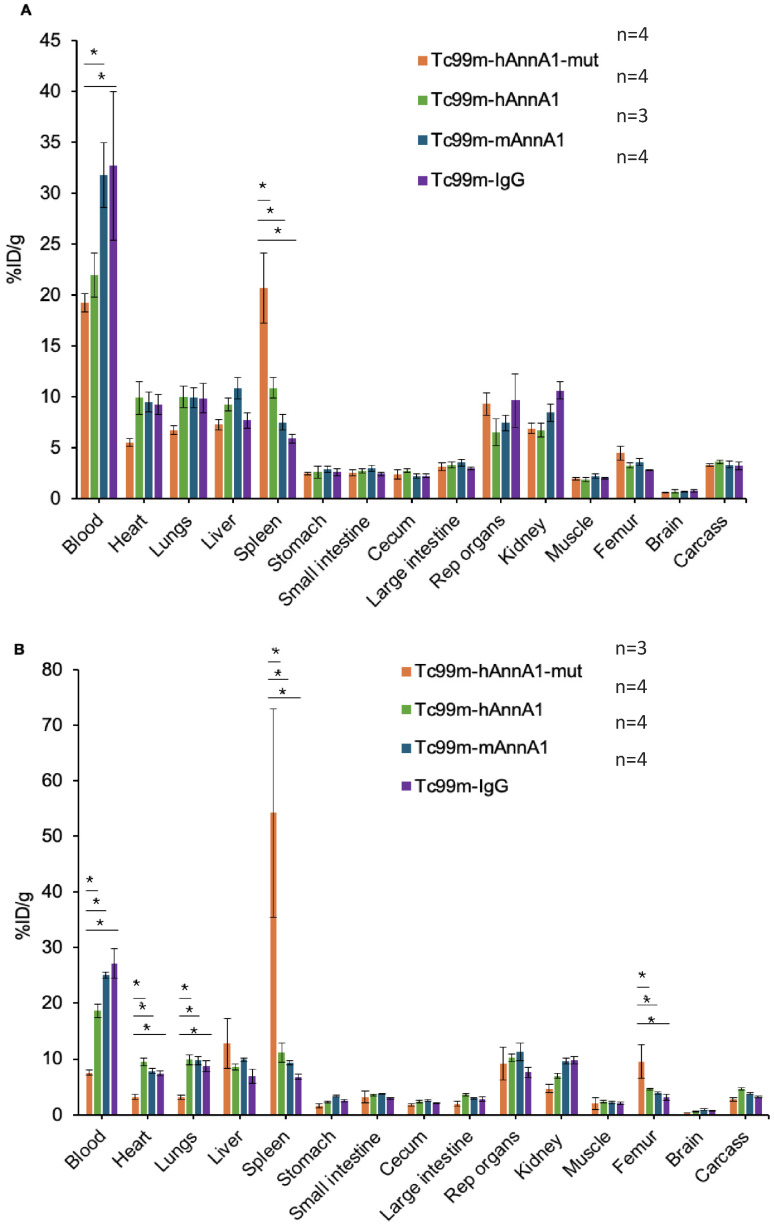
Differential uptake of AnnA1 antibody in different mice strains. Bar graphs showing biodistribution of antibody conjugated at 1:1 (HYNIC:Ab) and after 24 h of IP Dosing in (**A**) FVB mice (1:1 HYNIC:Ab) with hAnnA1, hAnnA1-mut, mAnnA1, and IgG antibody. (**B**) C57BL/6 mice with hAnnA1, hAnnA1-mut, mAnnA1, and IgG antibody. * *p* < 0.05.

An accurate preclinical model is needed to study the therapeutic efficacy of any humanized monoclonal antibody for drug delivery to the tumor sites. In the present study, we IP injected (conjugation ratio 1:1 HYNIC:Ab) FVB and BALB/c nude mice strains with Tc-99m-HYNIC-hAnnA1-mut, Tc-99m-HYNIC-hAnnA1, Tc-99m-HYNIC-mAnnA1, and Tc-99m-HYNIC-IgG and evaluated antibody biodistribution after 24 h. As shown in Figure 4A, the biodistribution at 24 h after IP dosing (1:1 HYNIC:Ab) with Tc-99m-HYNIC-hAnnA1, Tc-99m-HYNIC-hAnnA1-mut, Tc-99m-HYNIC-mAnnA1, and Tc-99m-HYNIC-IgG antibody in FVB mice showed that the spleen had significantly higher uptake of Tc-99m-HYNIC-hAnnA1-mut (20.7 ± 3.5 %ID/g) compared with Tc-99m-HYNIC-hAnnA1 (10.9 ± 1 %ID/g), Tc-99m-HYNIC-mAnnA1 (7.5 ± 0.7 %ID/g), and Tc-99m-HYNIC-IgG (5.9 ± 0.4 %ID/g) (*p* < 0.05). Both Tc-99m-HYNIC-hAnnA1-mut (19.2 ± 0.9 %ID/g) and Tc-99m-HYNIC-hAnnA1 (21.9 ± 2.2 %ID/g) had lower blood levels, but the levels were not statistically different (*p* > 0.05) compared with Tc-99m-HYNIC-mAnnA1 (31.8 ± 3.2 %ID/g) and IgG (32.7 ± 7.3 %ID/g). As shown in Figure 4B, biodistribution at 24 h after IP Dosing (1:1 HYNIC:Ab) with Tc-99m-HYNIC-hAnnA1, Tc-99m-HYNIC-hAnnA1-mut, Tc-99m-HYNIC-mAnnA1, and Tc-99m-HYNIC-IgG antibody using C57BL/6 mice demonstrated significantly higher spleen uptake of Tc-99m-HYNIC-hAnnA1-mut (54.2 ± 18.8 %ID/g) compared with Tc-99m-HYNIC-hAnnA1 (11.2 ± 1.7 %ID/g), Tc-99m-HYNIC-mAnnA1 (9.4 ± 0.4 %ID/g), and Tc-99m-HYNIC-IgG (6.8 ± 0.4 %ID/g) (*p* < 0.05). The femur had a notably greater uptake for Tc-99m-HYNIC-hAnnA1-mut (9.5 ± 3 %ID/g) compared with Tc-99m-HYNIC-hAnnA1 (4.6 ± 0.2 %ID/g), Tc-99m-HYNIC-mAnnA1 (3.9 ± 0.3 %ID/g), and Tc-99m-HYNIC-IgG (3.1 ± 0.6 %ID/g). The blood had significantly lower levels for Tc-99m-HYNIC-hAnnA1-mut (7.6 ± 0.4 %ID/g) compared with Tc-99m-HYNIC-hAnnA1 (18.7 ± 1.2 %ID/g), mAnnA1 (25.1 ± 0.5 %ID/g), and Tc-99m-HYNIC-IgG (27.2 ± 2.6 %ID/g) (*p* < 0.05). As shown in Figure 4, the amount of Tc-99m-HYNIC-hAnnA1-mut uptake at a 1:1 conjugation ratio, and IP injection in the liver and spleen in BALB/c nudes were comparable to the levels in C57BL/6 mice. There are no other remarkable differences in the level of uptake for other internal organs studied when comparing all strains of mice. These results show a drastically elevated uptake of Tc-99m-HYNIC-hAnnA1-mut in the liver of C57BL/6 and BALB/c nude mice compared with other Tc-99m-labeled antibodies. This could suggest that these two strains of mice were more sensitive to the underlying antibody sequence modifications as opposed to the increased sensitivity of FVB to surface modifications.

### 3.5. In Vitro Incubation Studies with Blood Sera from Various Sources

**Figure 5:**
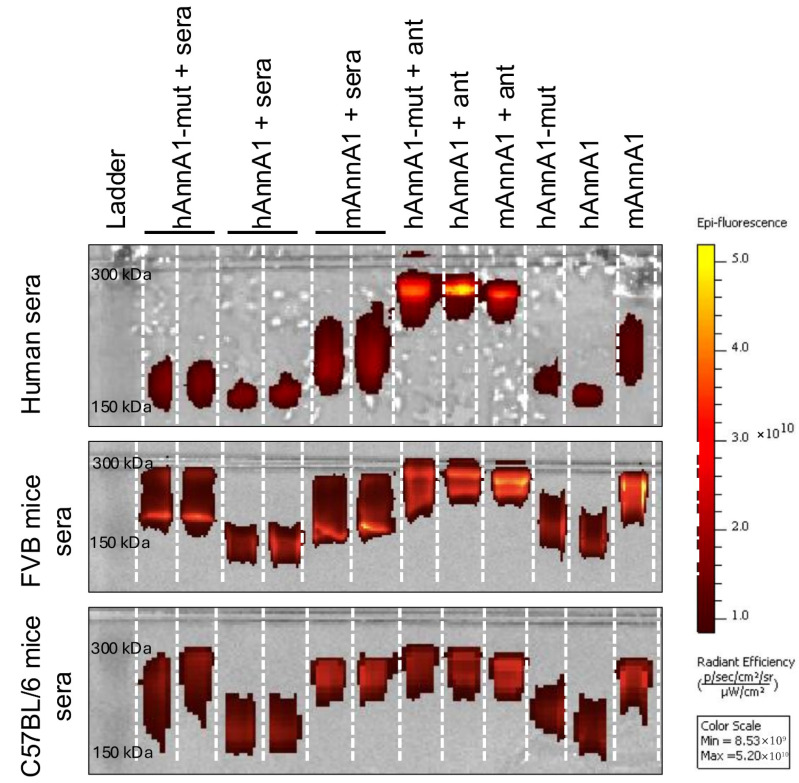
Serum binding assay for AnnA1 antibody binding with human and mice sera. Gel electrophoresis of human (**top**), FVB (**middle**), and C57BL/6 (**bottom**) mice blood sera incubated with hAnnA1-mut, hAnnA1, or mAnnA1 antibodies. The ladder is on the leftmost lane, spanning from 150 to 300 kDa. Ag = antigen or AnnA1 antibody target. Antibodies incubated with antigen (positive control) are in lanes 8–10. Antibody-only lanes (negative control) are in lanes 11–13. An upward shift compared with the negative control lanes represents binding to antibodies.

Given that complement proteins in the blood can opsonize foreign pathogens or proteins, leading to removal by the liver or spleen, we wanted to test whether the increased uptake in these organs was related to the binding of hAnnA1-mut to proteins within blood sera. Figure 5 illustrates gel electrophoresis data for the various antibodies incubated in sera from FVB mice, C57BL/6 mice, and humans. These results showed that the hAnnA1-mut antibody was shifted upwards in the gel because of the higher molecular weight of sera proteins bound to the hAnnA1-mut. Proteins from human sera (top lanes) had negligible binding to any of the antibodies. The incubation of hAnnA1-mut antibody with sera from FVB (middle lanes) and C57BL/6 (bottom lanes) both resulted in an upward shift compared with hAnnA1 and mAnnA1 antibodies alone with very low to no binding when comparing the signal intensity of these lanes. The antibody–protein conjugates from lanes where the antibody was incubated with mouse sera are estimated to lie around 250 kDa based on the 150 kDa and 300 kDa ladder. The positive control lanes with the AnnA1 antigen (~12 kDa, abbreviated Ant in Figure 5) were also around the same position, suggesting the antigens binding to hAnnA1-mut, hAnnA1, and mAnnA1 were complexed together. Based on the difference between the conjugates (antibody-serum protein/antigen) and the antibody-only negative control lane, it could be approximated that the sera proteins(s) attaching to hAnnA1-mut antibody were <100 kDa as determined by the upward shift of the bands. These gel electrophoresis results suggested that sera protein binding to antibodies likely contributed to increased in vivo RES uptake as the hAnnA1 antibody did not have an upward shift in the gels, which indicated no binding of sera proteins.

### 3.6. Histopathology Liver and Spleen Analysis

**Figure 6:**
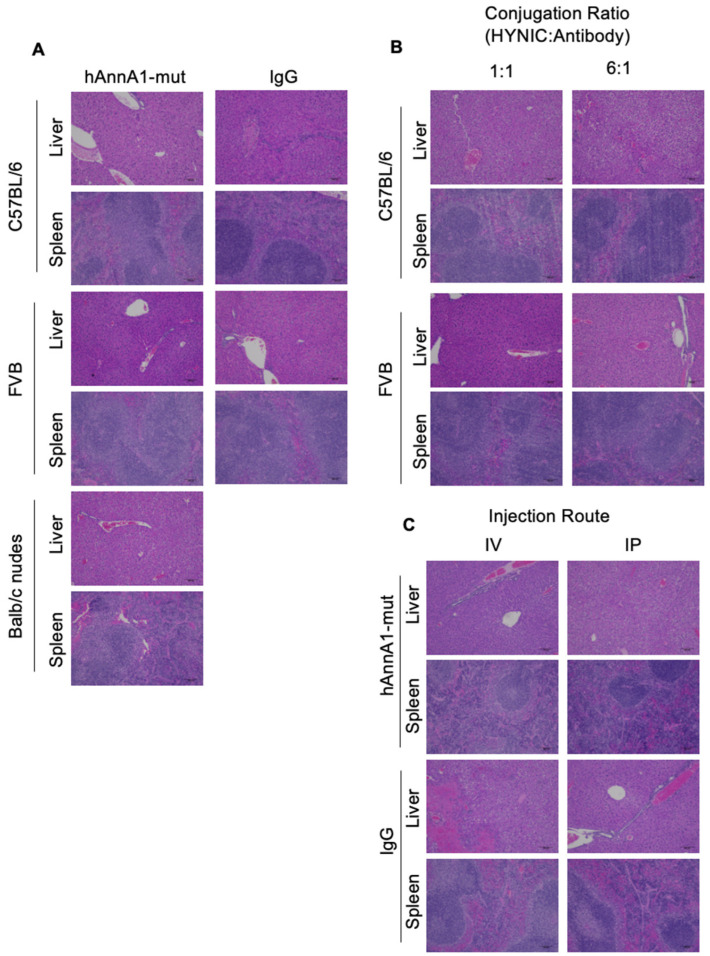
Histology slides of liver and spleen samples. (**A**) Comparison of hAnnA1-mut and IgG antibodies in C57BL/6, FVB, and BALB/c nudes mice. (**B**) Comparison between 1:1 and 6:1 HYNIC:hAnnA1 antibody conjugation ratio in C57BL/6 and FVB mice. (**C**) Comparison between IV and IP injection routes of hAnnA1 and IgG antibody in BALB/c nude mice.

H&E analysis (Figure 6) was performed for the liver and spleen samples comparing hAnnA1-mut vs. IgG of C57BL/6, FVB, and BALB/c nude mice, hAnnA1-mut 1:1 vs. 6:1 (HYNIC:Ab) conjugation comparison of liver and spleen samples for C57BL/6 and FVB mice, and hAnnA1-mut and IgG1 for IV vs. IP injection routes. The results showed that mild hepatitis and increased extramedullary hematopoiesis were present amongst various types of treatment conditions regardless of antibody, conjugation ratio, or injection route. This suggests that the increased liver and spleen uptake of hAnnA1-mut did not result in more prominent histopathological damage to the tissues compared with the isotype control (IgG), non-mutated hAnnA1, or mAnnA1.

## 4. Discussion

Monoclonal antibodies have become more commonly used for the treatment of various cancers and autoimmune diseases. Novel chimeric humanized or fully humanized Ab specific to previously validated targets is being developed to discover new therapeutic avenues. One application is antibody-drug conjugates that combine the advantages of highly specific targeting with potent killing effects to achieve more effective elimination of cancer cells [21]. The drugs are covalently attached to the antibody via a chemical linker, and alterations may have similar unwanted RES uptake effects. Hepatotoxicity and elevated liver enzymes have been reported for antibody-drug conjugates in breast cancer treatment [22]. FcγR-mediated internalization may play a role in clearing these complexes from circulation. It has been found that physiochemical properties of Ab, such as the amino acid sequence of the Fc region or the glycosylation profile, may influence FcγR binding affinity [23]; therefore, RES toxicity could be influenced by how many drug molecules are attached and to what location on the antibody. Antibodies have also been linked to nanoparticles for both imaging and treatment purposes, but their size may contribute to unwanted rapid scavenging by RES and poor penetration by endothelial cell barriers. Targeting the antibody-nanoparticle conjugate to caveolae was shown to avoid RES uptake while achieving rapid delivery to the target [24].

The validation of antibodies in pre-clinical settings is an important step of the FDA approval process to implement in clinics. Preparing a sufficient amount of high-quality antibodies that maintain their structural and chemical integrity for pre-clinical studies remains a challenge in many cases due to an abundance of steps that must be accurately followed [25]. Alterations in glycosylation profiles in marketed therapeutic antibodies are not uncommon, shown to have higher cleavage and aggregation rates [26]. There have not been any reports of adverse clinical effects or safety issues related to differences in specific glycosylation of antibodies because of their complexity, but analytical techniques are required, given that an antibody’s glycosylation profile can confer differing immune activation [27]. It has also been known that small differences in manufacturing, such as media composition, temperature, pH shifts, and expression systems, can result in significant differences in the glycosylation of biosimilars, which makes it even more vital to characterize their profiles [28]. The current studies with hAnnA1-mut were completed before we had knowledge about the Fc terminal amino acid sequence error, and we initially thought the spleen and liver uptake might have been related to the difference in glycosylation profile of the scaled-up production of hAnnA1.

Stromal fibroblasts, infiltrating immune cells, cytokines, and growth factors are some of the many components of the immune system that can affect both tumor development and clearance of foreign particles from the body [29]. The reticuloendothelial system (RES) is part of the immune system that consists of phagocytic cells in the liver and spleen that act as host defenses to remove pathogens and foreign material, including antibodies [30]. Previous studies have shown that RES organs are a common site of uptake for various monoclonal antibodies in immune incompetent mouse models. It was found that the immunodeficiency status of mouse models altered the in vivo fate of humanized antibodies to non-preferentially be sequestered in non-target organs, such as the bone and spleen. This spleen accumulation was blocked when Zr-89 radiolabeled humanized IgG1-traztuzumab, and cetuximab antibodies were co-injected with a 25-fold excess amount of isotype-matched anti-hapten humanized antibody [31]. Similarly, a study using Zr-89 radiolabeled anti-PD-L1 antibody showed rapid localization to the spleen and lymph nodes in PD-1/PD-L1 humanized mice, but the splenic uptake was reduced when mice were treated with clodronate liposomes that systemically depleted PD-L1 expressing phagocytic cells [32]. There also appeared to be a strong correlation between impaired binding of deglycosylated Zr-89 labeled trastuzumab radioimmunoconjugates targeting EGFR2 with truncated glycans and significantly decreased in vivo off-target uptake with a concomitant increase in tumoral accumulation that improved immuno-PET quality [33]. The deglycosylation of the CH2 domain of the IgG1 antibody transformed the native horseshoe-like configuration to a closed state, thus blocking access to Fc-gamma-receptor (FcγR). This suggests that Fc receptor engagement contributed to RES uptake. Another study also found that complement-dependent cell-mediated cytotoxicity and complement-dependent cell-mediated phagocytosis by immunological effector molecules mediated the clearance of target proteins with comparable kinetics and efficacy as FcγR-dependent mechanisms [34].

The current study supports that modifications to the constant regions of IgG1 can result in differences in liver and spleen uptake, as illustrated by the significantly higher uptake of Tc-99m-HYNIC-hAnnA1-mut of all spleen samples regardless of mouse strain, injection route, or molar conjugation ratio. These results are in alignment with another group that used the same hAnnA1-mut targeting the truncated Annexin A1 and revealed enhanced spleen uptake with PET/CT 7 days post-injection, comparing Zr-89-DFO-hAnnA1-mut with Zr-89-DFO-mAnnA1 [35]. High splenic uptake was seen in both immuno-competent and immuno-compromised mice with the highest in the BALB/c nude strain [35]. As shown in Figure 2, an increased molar conjugation ratio (HYNIC:Ab) from 1:1 to 6:1 increased the RES uptake of Tc-99m-HYNIC-hAnnA1-mut for both C57BL/6 and FVB mice. Using a 6:1 conjugation ratio means there are 6× more HYNIC attached to each antibody, causing a greater structural change to the antibody for it to be more likely to be recognized as foreign. Our data suggest that even minor modifications increased the RES uptake for an antibody with an altered constant region structure (hAnnA1-mut) but had no significant impact on the distribution of the antibody that had no changes to their constant region (hAnnA1); therefore, minor changes to the antibody may have been recognized by the complement system and accumulated in the liver or spleen for its removal. RES, which includes macrophages of the liver and spleen, is the main route for phagocytic removal of unwanted circulatory components [30]. The liver and spleen levels of the correct sequence of Tc-99m-HYNIC-hAnnA1 were not different from Tc-99m-HYNIC-mAnnA1 and Tc-99m-HYNIC-IgG1, indicating RES uptake should not be a problem for future uses of hAnnA1.

The antibody-to-chelator molar ratio is a characteristic of the conjugation process that attaches radioisotope chelators to antibodies. The conjugation ratio plays an important role in determining the maximum level of radioactivity that can be bound to the antibody, which can influence both imaging and therapeutic applications of radiolabeled antibodies. Previous studies have found that a higher degree of antibody labeling alters the pharmacokinetics by reducing the quality of images for antibody-fluorophore conjugates and decreasing the therapeutic index for antibody-drug conjugates because of the faster rates of clearance [18,19]. More specifically, doubling the drug-to-antibody conjugation ratio resulted in at least a threefold increase in the % injected dose/g localized to the liver [20]. These observations agree with our study, where a higher conjugation ratio in FVB mice (Figure 2B) resulted in significantly higher uptake in the liver, particularly for hAnnA1-mut with additional mutations. For our purposes, the 1:1 conjugation molar ratio represented a minor change while the 6:1 molar ratio represented a slightly greater change, keeping in mind that the chelator conjugation process is random through lysine residues that are chemically accessible in the antibody structure, and a typical IgG1 antibody has approximately 85 total lysine residues. Of note, the hAnnA1-mut had no lysine residues in the random twenty-four amino acids at the Fc terminus, while the correct sequence of hAnnA1 had two lysine residues in the sixteen amino acids at the Fc terminus.

Differences in RES uptake were also apparent between mouse strains (Figure 4A vs. Figure 4B vs. Figure 3) and injection routes (Figure 3). Of all the immune-competent mice used, FVB mice showed the lowest level of hAnnA1-mut uptake in the liver or spleen and the highest amount in the blood. A study found that FVB mice expressed significantly less GARP and LAP, which is the downstream effector molecule of Foxp3 transcription factor for the normal production and function of regulatory T cells [36]. Thus, the lower amount of regulatory T cells of FVB may be correlated with reduced RES uptake. The IV injection route had a significantly higher level of liver and spleen uptake of hAnnA1-mut compared with IP injection. IV injection directly enters the bloodstream as opposed to IP injection, which diffuses more slowly into the blood. This is supported by prior studies where the spleen is the preferential site of antibody accumulation [37]. Additionally, IP injection had a higher uptake in the reproductive organs, which are in close proximity to the site of administration. It has been reported that the spleen is the third most important site for monoclonal antibody degradation after the gut and liver, contributing to 3.6% of whole-body disposal [37]. Although there is no damage to the tissues of the liver and spleen, according to histopathology results, levels need to be monitored when administering radioimmunotherapy. When choosing the best therapeutic option, it is crucial to consider extraneous factors that may impact toxicity. A limitation of this study is that only three mice strains were assessed, and different strains may show various combinations/amounts of complement factors that impact RES uptake. Nevertheless, the diversity in strains used is adequate in showing the role of hAnnA1-mut in increased RES uptake. The %ID/g of various organs may vary slightly, but the error associated with the scale for tissue weighing and the gamma counter readings used to measure the syringes are negligible.

Mouse models are initially required to evaluate the effectiveness of cancer treatments as they exhibit high tumor growth, short reproductive cycles, and genetic modifications are easy. The current challenge is translating results from bench to clinic because of the high failure rates observed in human clinical trials after promising results were obtained in mouse studies [38]. Syngeneic tumor models have been the most used approach, where mouse cancer cells are transplanted into an immunocompetent mouse. Although this model is easy to set up and tumor development is rapid, it lacks characteristics that make this effective for human translation. Cancer research has been progressing towards patient-derived xenograft models where primary patient tumors are implanted into immune-competent mouse models. This better recapitulates the human tumor microenvironment, reflects the complexity of growth, and has a high heterogeneity of cells, allowing for a more accurate translation [39].

The gel electrophoresis results (Figure 5) were consistent with the 24 h biodistribution data as hAnnA1-mut exhibited binding to a protein in the incubated sera in a similar manner as the positive control lane with AnnA1 antigen. This could be due to complement opsonization, leading to subsequent removal by the liver and spleen as part of our innate immune system. Thus, the mutated portion of the antibody located at the Fc constant region was recognized as foreign and targeted for destruction through opsonization, lysis, or inflammation [40]. Out of the three pathways through which the complement system is activated, the classical pathway is the most likely route for recognizing antigen-antibody immune complexes. When C1q binds to the complex, it activates C1r and C1s, which cleaves C4 and C2. C1q is ~460 kDa and composed of 18 polypeptide chains, which is below the estimated size of the bound protein of <100 kDa [41]. The human IgG1 subclass served as an appropriate negative control for our tested antibodies in our mouse models. Human IgG1 has non-specific binding and binds to mouse FcγR with similar binding strengths to human ortholog receptors [42]. A study that altered human IgG with triple mutations that prevented the binding of the Ab to FcγR exhibited lower uptake in the liver and improved tumor targeting [43]. This suggests that, more generally, minor modifications to the Fc region could change their binding characteristics (reduced Fc receptor binding), potentially in a favorable way. Another study showed that framework mutations are consistent between individuals where the function could be inferred from sequence [44]; therefore, the Fc mutations of hAnnA1 possibly changed the conformation of the protein to activate the complement cascade through FcγR expressing innate immune effector cells resulting in increased RES uptake. Non-lymphoid organs have been known to have FcγR expression on resident macrophages, such as Kupffer cells [45]. Our gels also showed that both FVB and C57BL/6 mice sera exhibited binding to hAnnA1-mut, but human sera lacked such binding, which could indicate reduced RES sensitivity to antibody alterations. The gel electrophoresis assay is limited in determining what exact proteins and signaling pathways are involved in antibody binding. Future studies can consider Western blot and mass spectrometry to determine the exact mechanism behind increased RES uptake of hAnnA1-mut.

## 5. Conclusions

Our study reinforces the importance of using the correct protein sequences with proper handling to maintain the integrity of antibodies to prevent unwanted RES uptake. The present study indicated that modifications to the hAnnA1 protein sequence in the Fc region led to higher liver and spleen uptake and lower blood levels, suggesting hAnnA1-mut antibody’s recognition by the innate immune system and clearance. Higher levels of complement protein binding in the Fc region likely explain the present study’s findings. The comparable levels in the liver and spleen of the non-mutated antibody relative to IgG and mAnnA1 support the use of hAnnA1 for further preclinical studies in preparation for clinical translation. This suggests that modifications to antibodies need to be avoided to prevent uptake in non-target organs.

## Figures and Tables

**Table 1:** Antibody characteristics comparing the length of hAnnA1 and hAnnA1-mut (aa = amino acid, bp = base pair) between the coding nucleotide and translated amino acid.

Characteristic	hAnnA1	hAnnA1-mu
Coding nucleotide	1338 bp	1362 bp
Translated amino acid	445 aa	453 aa

## Data Availability

Data is contained within the article.
